# Correlates of sport participation in adults with long-standing illness or disability

**DOI:** 10.1136/bmjsem-2015-000003

**Published:** 2015-06-03

**Authors:** Neil Heron, Frank Kee, Margaret E Cupples, Mark A Tully

**Affiliations:** 1 Department of General Practice and Primary Care, Queen's University Belfast, Belfast, UK; 2 Centre for Public Health, Queen's University Belfast, Belfast, UK; 3 UKCRC Centre of Excellence for Public Health (NI), Queen's University Belfast, Belfast, UK

**Keywords:** Correlates, Sport participation, Long-term illness or disability, Health inequalities

## Abstract

**Background:**

Little is known about why people with a long-standing illness/disability are less likely to participate in sport than others. This study aimed to identify for the first time sport participation levels and their correlates among Northern Ireland (NI) adults who report a long-standing illness/disability.

**Method:**

Using data collected in the Continuous Household Survey, an annual survey of a random sample of the NI population, during 2007–2011, we examined responses for the total sample, those with a long-term illness/disability and those with no long-term health issues. We conducted univariate binary regression analysis for the whole sample and for those with a long-standing illness or disability, using sport participation as the dependent variable, and then carried significant variables into a multivariate analysis.

**Results:**

The sample included 13 683 adults; 3550 (26%) reported a long-term illness or disability. Multivariate analysis showed that, for the total sample and for those with a long-standing illness or disability, sport participation correlated positively with being male, aged <56 years, having a household car/van, health being ‘fairly good’/‘good’ in the previous year, doing work and living in an urban location. Also, for those with a long-standing illness or disability, being single and less socioeconomically deprived correlated positively with sport participation.

**Conclusions:**

The findings suggest that more focused efforts may promote sport participation for people with a long-standing illness or disability who are female, older, not working, living rurally, married/cohabiting, socioeconomically deprived and report having had poor health in the past year. Our findings should inform public health policy and help in developing initiatives to support sport participation and reduce health inequalities.

**What is already known**

- People with a long-term illness or disability are less likely to participate in sport than the general population.
- People with a long-term illness or disability have poorer health than the general population and their failure to participate in sport further exacerbates the known health inequalities.

## Introduction

As the age of the UK population slowly increases, so does the proportion of the population living with a disability or long-term health condition.^1^ Despite there being clear evidence of the benefits of physical activity for the whole population,^2^ people with disabilities/long-term illnesses are less likely to be active, meet physical activity recommendations or participate in sport.^3–7^ There is therefore a need for further information on the effects of environmental and societal barriers in sport participation for people with disabilities/long-term conditions,^8–10^ to inform policies aimed at increasing their participation in sport and physical activity.^11^

Promoting physical activity^3^ among the whole population, including those with a disability/long-term health problem, is important. Regular physical activity improves mental well-being and contributes to the management of chronic conditions,^2^ ^12^ including retardation of the functional decline often associated with disabling conditions.^13^ Guidelines exist for physical activity prescription in those with various disabling/long-term conditions,^14^ ^15^ but despite this people with a disability are half as likely to be active than the general population.^4^ ^7^ There is a need to tackle this health inequality.^2^ ^16–18^ Physical inactivity among those who are disabled may lead to secondary health conditions, engendering further health inequalities. People with a physical disability are more likely to be obese,^13^ ^19^ ^20^ with increased risk of chronic, non-communicable diseases.^19^ ^21^ A paradigm shift from disability prevention to prevention of secondary conditions by increasing physical activity would help to address health inequalities in this disadvantaged population.^9^

Reasons for low levels of participation in physical activity and sport in those with a disability/long-standing illness are complex and thought to include social, cultural and environmental factors.^7^ ^22^ Trost *et al*^23^ identified biological, psychological and social factors as important correlates of physical activity in able-bodied adults. Bodde *et al*^6^ described similar correlates of moderate-vigorous physical activity participation in adults with intellectual disabilities. Rimmer *et al*,^7^ through focus groups in the USA, identified major barriers and facilitators associated with disabled people's participation in fitness and recreation programmes. However, no previous studies have reported the correlates of physical activity and sport participation in adults with a long-term illness/disability in the context of the UK National Health Service, whereby individuals with a long-standing illness/disability receive most of their healthcare free at the point of delivery or in NI, where public health and social care services are integrated. The purpose of this study was to identify levels of sport participation, based on self-report, and the correlates of sport participation in those reporting long-standing illness/disability within the NI adult population.

**What this paper adds**

- This paper identifies new correlates of sport participation for UK adults with a long-standing illness or disability.
- This information will allow public health professionals to identify new targets to help address some of the known health inequalities in those with a long-standing illness or disability.
- People with a long-standing illness/disability who report their health as ‘not good’ in the previous 12 months need targeted physical activity and sport participation counselling. This knowledge should be utilised by general practitioners and primary care health professionals.

## Methods

### The Continuous Household Survey (CHS)

The current study data were taken from the Continuous Household Survey (CHS). No ethical approval was required as the study involved secondary analysis of anonymised information. CHS is a large continuous survey carried out annually in NI^24^ since 1983. It is designed, conducted and analysed by the Central Survey Unit (CSU) of the NI Statistics and Research Agency (NISRA) and provides information on a wide range of social and economic issues. A stratified random sample of 4500 addresses is drawn each year from the ‘Pointer’ list of domestic addresses, based on geographical areas attributable to specified household postcodes. Pointer is the address database for NI and is maintained by the Land and Property Services.

The survey questionnaire consists of a household interview and an individual interview with each person aged 16 years and over in the selected households. The household and individual questionnaires consist of core items that are included each year as well as other items, which are set each year following consultation with other government departments. This study analysed the data available from the questionnaire responses to the set questions.

### The study sample

Pooling 2007–2011 data from CHS yielded a sample of 13 683 adults, with 3550 (26%) reporting a long-standing illness or disability in response to the question:

“Do you have any long-standing illness, disability or infirmity? By long-standing I mean anything that has troubled you over a period of time or that is likely to affect you over a period of time.”

Summary demographic data for the sample population are in keeping with those of the NI general population.^25^

### Data management and analysis

On the basis of whether or not they reported having participated in sport at least once in the last year, individuals were categorised into those who participated in sport and those who did not. Various self-reported independent predictor variables were extracted from the data set (tables 1 and 2). Using the NI Multiple Deprivation Measure (MDM)^26^ derived from respondents’ home postcodes, individuals were categorised into deciles of socioeconomic deprivation.

**Table 1:** Descriptive statistics for the full study cohort, those with a long-term illness or disability and those with no long-term health problems and comparisons of distribution of categorical variables

Variable	Full cohort		Long-term illness/disability		No long-term illness/disability		p Value*
	N	Per cent	N	Per cent	N	Per cent	
Sex							0.020
Male	5754	42.1	1425	40.1	4324	42.8	
Female	7905	57.9	2125	59.9	5774	57.2	
Age group (years)							<0.001
16–25	1369	10	99	2.8	1269	12.6	
26–35	2266	16.6	247	7.0	2017	20.0	
36–45	2540	18.6	469	13.2	2070	20.5	
46–55	2380	17.4	597	16.8	1780	17.6	
56–65	2165	15.9	776	21.9	1388	13.7	
66–75	1738	12.7	752	21.2	985	9.8	
>75	1201	8.8	610	17.2	589	5.8	
Marital status							<0.001† (testing grouped variables)
Married	7455	54.6	1762	49.6	5687	56.3	
Cohabiting	777	5.7	92	2.6	683	6.8	
Single	2911	21.3	597	16.8	2311	22.9	
Widowed	1276	9.3	617	17.4	659	6.5	
Divorced	703	5.1	288	8.1	415	4.1	
Separated	521	3.8	191	5.4	330	3.3	
Same sex couple	16	0.1	3	0.1	13	0.1	
Religion							<0.001
Catholic	5527	40.5	1367	38.5	4157	41.2	
Protestant	7184	52.6	1972	55.5	5209	51.6	
Other/none	811	5.9	191	5.4	618	6.1	
Education							<0.001
Degree	1949	14.3	180	5.1	1768	17.5	
All other qualifications	6695	49.0	1250	35.2	5442	53.9	
No qualifications	2835	20.8	1065	30.0	1768	17.5	
Sport participation							<0.001
Yes	6486	47.5	868	24.5	5615	55.6	
Days/week in sport							<0.001
0	1600	11.7	307	8.6	1293	12.8	
1	1679	12.3	227	6.4	1451	14.4	
2	1192	8.7	139	3.9	1052	10.4	
3	868	6.4	91	2.6	777	7.7	
4	378	2.8	26	0.7	351	3.5	
5	332	2.4	32	0.9	300	3.0	
6	114	0.8	6	0.2	108	1.1	
7	305	2.2	39	1.1	266	2.6	
Sport (min/week)							0.802
0–30	4321	96.8	593	98.2	3727	96.6	
>30, <60	57	0.13	4	0.7	53	1.4	
60–90	16	0.4	1	0.2	15	0.4	
>90–150	44	1.0	4	0.7	40	1.0	
>150	27	0.6	2	0.3	25	0.6	
Sports club member							<0.001
Yes	2757	20.2	389	11.0	2365	23.4	
Health last year							<0.001
Good	7673	56.2	433	12.2	7238	71.7	
Fairly good	3706	27.1	1212	34.1	2491	24.7	
Not good	2275	16.7	1904	53.6	368	3.6	
Current smoker							
Yes	3210	44.9	974	47.0	2234	44.1	0.073

*χ^2^ Tests were used to compare the groups: those with a long-term illness to those with no long-term illness.

†Footnotes groupings for χ^2^ tests—married/cohabiting v single v widowed/divorced/separated/same sex couple.

**Table 2:** Socioeconomic and related data compared between the full study cohort, those with a long-term illness or disability and those with no long-term health problems

Variable	Full cohort		Long-term illness/disability		No long-term illness/disability		p Value*
	N	Per cent	N	Per cent	N	Per cent	
Household car							<0.001
Yes	10 964	80.3	2384	67.2	8573	84.9	
Paid work/week							<0.001
Yes	6586	48.2	584	16.5	5998	59.4	
Unpaid work last week for own business?							0.040
No	6402	99.1	2865	99.4	3531	98.9	
Unpaid work last week for any family business?							0.777
No	6383	99.7	2858	99.8	3519	99.7	
NSSEC 3†							<0.001
Managerial/professional Intermediate	3855	28.2	698	19.7	3154	31.2	
Routine/manual	2905	21.3	678	19.1	2225	22.0	
Never worked	5639	41.3	1857	52.3	3780	37.4	
Full time education	509	3.7	225	6.3	283	2.8	
Not classified	751	5.5	92	2.6	656	6.5	
Benefits							
Yes	7792	57.0	2986	84.1	4799	47.5	<0.001
Residence							
Urban	8827	64.6	2355	66.3	6465	64.0	0.072
NUTS3‡							<0.001
Belfast	2144	15.7	630	17.7	1514	15.0	
Outer Belfast	3019	22.1	779	21.9	2236	22.1	
East of NI	3343	24.5	797	22.5	2540	25.2	
North of NI	2049	15.0	542	15.3	1506	14.9	
West+South NI	3016	22.1	786	22.1	2230	22.1	
Household internet							
Yes	8602	63.0	1582	44.6	7013	69.4	<0.001
Personal internet							
Yes	8551	62.6	1471	41.4	7075	70.1	<0.001
Do you have internet access?							<0.001
Yes	7791	91.1	1235	84.0	6551	92.6	
MDM deciles							<0.001
1	1210	8.9	447	12.6	763	7.6	
2	1274	9.3	408	11.5	864	8.6	
3	1280	9.4	385	10.8	892	8.8	
4	1449	10.6	381	10.7	1066	10.6	
5	1422	10.4	385	10.8	1037	10.3	
6	1407	10.3	356	10.0	1051	10.4	
7	1449	10.6	319	9.0	1129	11.2	
8	1488	10.9	336	9.5	1151	11.4	
9	1385	10.1	276	7.8	1108	11.0	
10	1295	9.5	257	7.2	1037	10.3	

*χ^2^ tests were used to compare the groups: those with a long-term illness to those with no long-term illness.

†NSSEC3—marker of social class.

‡NUTS3—Northern Ireland is split up into 5 different geographical areas.

NI, Northern Ireland.

All analyses were conducted using the Statistical Package for Social Sciences (SPSS), V.21.0 Software for Windows (SPSS Inc, Chicago, USA). χ^2^ Tests were used to identify differences in the distribution of variables between those with a long-standing illness/disability and those without (tables 1 and 2). Univariate and multivariate analyses were carried out for those reporting a long-standing illness/disability (table 3) and the full study cohort (table 4).

**Table 3:** Univariate and multivariate logistic regression analyses for those with a long-term illness or disability

Variable	Univariate analysis			Multivariate analysis		
	OR	CI	p Value	OR	CI	p Value
Sports clubs membership?						
Yes	13.080	10.235 to 16.717	<0.001	9.759	6.825 to 13.955	<0.001
Sex						
Male	1.614	1.383 to 1.883	<0.001	1.667	1.298 to 2.141	<0.001
Age groups (years)						
16–35	10.727	7.586 to 15.168	<0.001	5.212	2.586 to 10.505	<0.001
36–55	4.856	3.587 to 6.576	<0.001	2.331	1.236 to 4.395	0.009
56–75	2.229	1.645 to 3.021	<0.001	1.170	0.675 to 2.027	0.577
76 and over	Ref	–	–	Ref	–	–
Marital status						
Married or cohabiting	0.794	0.650 to 0.969	0.024	0.618	0.426 to 0.897	0.011
Widowed, divorced, separated or same sex couple	0.397	0.313 to 0.503	<0.001	0.859	0.583 to 1.265	0.442
Single	Ref	–	–	Ref	–	–
House car/van?						
Yes	2.447	2.033 to 2.946	<0.001	1.644	1.197 to 2.257	0.002
Highest qualifications						
Degree or higher	7.625	5.422 to 10.723	0.000	1.573	0.875 to 2.828	0.130
All other qualifications	3.933	3.199 to 4.836	0.000	2.181	1.614 to 2.948	<0.001
No qualifications	Ref	–	–	Ref	–	–
Paid or unpaid work in the past 7 days?						
Yes	4.476	3.714 to 5.393	<0.001	1.475	1.026 to 2.120	0.036
Benefits?						
Yes	0.327	0.270 to 0.396	<0.001	0.920	0.631 to 1.339	0.662
Urban or rural?						
Rural	0.679	0.572 to 0.805	<0.001	0.716	0.525 to 0.975	0.034
NUTS3						
Belfast	1.396	1.096 to 1.777	0.007	1.632	1.058 to 2.519	0.027
Outer Belfast	1.614	1.287 to 2.024	<0.001	1.308	0.873 to 1.961	0.193
East of NI	0.890	0.699 to 1.133	0.346	0.915	0.623 to 1.346	0.653
North of NI	0.825	0.628 to 1.084	0.167	0.915	0.600 to 1.396	0.680
West+South NI	Ref	–	–	Ref	–	–
Over the last year, has your health on the whole been						
Good	3.044	2.432 to 3.809	<0.001	2.300	1.581 to 3.347	<0.001
Fairly good	1.793	1.512 to 2.126	<0.001	1.567	1.197 to 2.050	<0.001
Not good	Ref	–	–	Ref	–	–
Do you smoke?						
Yes	0.853	0.701 to 1.039	0.115			
Personal internet access?*						
Yes	3.377	2.873 to 3.968	<0.001	1.302	0.983 to 1.723	0.066
MDM quintiles						
1	0.459	0.360 to 0.583	<0.001	0.334	0.158 to 0.708	0.004
2	0.433	0.337 to 0.555	<0.001	0.362	0.192 to 0.680	0.002
3	0.485	0.379 to 0.621	<0.001	0.297	0.157 to 0.562	<0.001
4	0.627	0.490 to 0.802	<0.001	0.415	0.228 to 0.757	0.004
5	Ref	–	–	Ref	–	–

*Personal internet access combines the 3 groups (household, personal and any internet access) into 1 variable.

MDM, Multiple Deprivation Measure; NI, Northern Ireland.

**Table 4:** Univariate and multivariate logistic regression analyses for the full study population

Variable	Univariate analysis			Multivariate analysis		
	OR	CI	p Value	OR	CI	p Value
Sports club membership?						
Yes	13.464	11.887 to 15.250	<0.001	9.852	6.891 to 14.085	<0.001
Sex						
Male	1.721	1.607 to 1.843	<0.001	1.574	1.389 to 1.783	<0.001
Age groups (years)						
16–35	13.694	11.485 to 16.328	<0.001	5.556	3.705 to 8.331	<0.001
36–55	6.552	5.525 to 7.770	<0.001	2.893	1.957 to 4.276	<0.001
56–75	2.632	2.210 to 3.134	<0.001	1.409	0.991 to 2.003	0.056
76 and over	Ref	–	–	Ref	–	–
Marital status						
Married or cohabiting	0.704	0.647 to 0.767	<0.001	0.934	0.789 to 1.105	0.425
Widowed, divorced, separated or same sex couple	0.30	0.268 to 0.336	<0.001	1.059	0.859 to 1.306	0.593
Single	Ref	–	–	–	–	–
House car or van?						
Yes	2.722	2.483 to 2.983	<0.001	1.307	1.102 to 1.551	0.002
Highest qualifications						
Degree or higher	8.912	7.798 to 10.184	<0.001	3.172	2.504 to 4.018	<0.001
All other qualifications	3.785	3.436 to 4.169	<0.001	1.872	1.605 to 2.184	<0.001
No qualifications	Ref	–	–	Ref	–	–
Any paid/unpaid work in last week?						
Yes	3.796	3.533 to 4.079	<0.001	1.178	1.011 to 1.373	0.036
Benefits?						
Yes	0.343	0.319 to 0.368	<0.001	1.081	0.932 to 1.253	0.304
Urban or rural?						
Rural	0.913	0.850 to 0.980	0.012	0.846	0.731 to 0.979	0.025
NUTS3						
Belfast	1.233	1.104 to 1.378	<0.001	1.394	1.118 to 1.737	0.003
Outer Belfast	1.378	1.246 to 1.525	<0.001	1.038	0.851 to 1.266	0.714
East of NI	1.034	0.937 to 1.141	0.508	1.073	0.898 to 1.283	0.436
North of NI	0.759	0.677 to 0.851	<0.001	0.695	0.568 to 0.850	<0.001
West+South NI	Ref	–	–	Ref	–	–
Health over the last year been:						
Good	5.142	4.608 to 5.738	<0.001	1.801	1.454 to 2.230	<0.001
Fairly good	2.460	2.182 to 2.772	<0.001	1.543	1.258 to 1.892	<0.001
Not good	Ref	–	–	Ref	–	–
Limiting long-standing illness						
Yes	0.258	0.237 to 0.282	<0.001	0.736	0.618 to 0.876	0.001
Do you smoke?						
Yes	0.719	0.655 to 0.790	<0.001	0.735	0.646 to 0.837	<0.001
Personal internet?*						
Yes	5.140	4.755 to 5.556	<0.001	1.779	1.469 to 2.154	<0.001
MDM quintiles						
1	0.418	0.374 to 0.468	<0.001	0.717	0.504 to 1.018	0.063
2	0.480	0.431 to 0.535	<0.001	0.685	0.519 to 0.905	0.008
3	0.571	0.513 to 0.636	<0.001	0.788	0.599 to 1.038	0.090
4	0.750	0.675 to 0.834	<0.001	0.891	0.691 to 1.148	0.372
5	Ref	–	–	Ref	–	–

*Personal internet access combines the three groups (household, personal and any access) into one variable.

MDM, Multiple Deprivation Measure; NI, Northern Ireland.

For the univariate and multivariate logistic regression analyses, the dependent variable was sport participation in the last year. A significance level p<0.1 within the univariate analysis was used to identify variables to be taken forward into the multivariate analysis.^27^ A statistical significance level of <0.05 was set for the multivariate analysis and multivariate logistic regression analyses were conducted on the whole study population and for those who reported having a disability or long-term illness in the past 12 months.

## Results

### Demographic characteristics

The total sample included 5754 males (42.1%), with representation from ages 16 to over 75 years (tables 1 and 2). Overall, 6486 (47.5%) reported having participated in sport in the last year. Only 16.7% said their health was not good in the previous 12 months; 44.9% were current smokers. Participants were from all areas of NI and from a range of socioeconomic categories: 5639 (41.3%) had routine/manual occupations, 80.3% reported having access to a car/van in the household, 57% were receiving state benefits and 63% had household internet access.

Significantly fewer of those *with* than *without* a long-standing illness/disability (tables 1 and 2) reported sport participation in the past year (5.6% v 24.5%, p<0.05). Among those who reported having participated in sport, people with a disability were less likely to be a current member of any sports club/organisation (11.0% v 23.4%). Those with a long-standing illness/disability included more females, older age groups and people not married or cohabiting. People with a disability were less likely to have a household car/van, have a degree as their highest educational qualification and less likely to be doing work (paid or unpaid) in the past 7 days than those not reporting a long-standing illness/disability. They were also more likely to be categorised in lower socioeconomic groups (NSSEC3 categories, deprivation income and MDM deciles), to receive state benefits, to report their health over the previous 12 months as ‘not good’ and not to have internet access, either in the household or on a personal basis.

No significant differences were found between those with a long-standing illness/disability compared to those with no long-term health issues in respect of their living in an urban or rural location, being a current smoker or doing unpaid work for any business that a relative owns.

### Univariate and multivariate logistic regression

Multivariate analysis for those with a limiting long-standing illness/disability showed positive associations between sport participation in the last year and current membership of any sports clubs/organisations (OR 9.759, 95% CI 6.825 to 13.955), male sex (OR 1.667, 95% CI 1.298 to 2.141), having a household car/van (OR 1.644, 95% CI 1.197 to 2.257) and being younger (aged <56 years compared to being >75 years) (table 3). Those who were married or cohabiting were less likely to participate in sport than those who were single. Having any qualification, except a degree or higher, compared to no qualifications was positively associated with sport participation, as was doing any work in the past 7 days. Living in a rural location compared to an urban location was negatively associated with sport participation. Having ‘good’ and ‘fairly good’ health (compared to ‘not good’ health) over the past 12 months was correlated with sport participation, and deprivation (measured by MDM quintiles) was negatively correlated.

Multivariate analysis (table 4) for the whole population showed significant associations for sport participation with current membership of sports clubs/organisations (OR 9.852, 95% CI 6.891 to 14.085), male sex (OR 1.574, 95% CI 1.389 to 1.783), having a household car/van (OR 1.307, 95% CI 1.102 to 1.551) and younger age (<56 years compared to being >75 years). Having any qualification compared to no qualification was positively associated with sport participation as was doing any work in the past 7 days. Living in a rural location compared to living in an urban environment was negatively correlated with sport participation. Other positive correlations included health over the past 12 months being ‘good’ and ‘fairly good’, compared to ‘not good’ and having personal internet access. Having a limiting long-standing illness, deprivation (measured by MDM quintiles) and currently smoking correlated negatively with sport participation.

## Discussion

This study has identified new information about correlates of sport participation among adults in NI who report long-standing illness/disability. Survey data have shown that people with a long-term illness/disability who were single rather than married/cohabiting and who were least socioeconomically deprived were more likely to have participated in sport during the previous year. Variables which were correlated positively with sport participation in both the full study sample and the subgroup with long-term illness/disability included being male, younger than 56 years, access to a household car/van, sports club/organisation membership, health being ‘fairly good’ or ‘good’ in the previous 12 months, doing paid or unpaid work in the past 7 days and living in an urban location. While sport participation in the full sample was positively associated with higher educational status, being a non-smoker and with having personal internet access, this association was not significant for those with long-term illness/disability.

### Comparison with previous literature

Our finding of 26% with a long-term illness/disability is lower than that reported for other areas of the UK: 41% and 43% of adult males and females in England report long-term illness.^28^ This may be partly explained by the fact that NI has a younger population than the other nations within the UK^1^ as well as by possible differences in the public's interpretation of such questions.

A strategy for the development of sport and physical recreation in NI^11^ is aiming to increase the number of people in membership of at least one sports club by 2014. The 2010 Sport and Physical Activity Survey (SAPAS) reported that 23% of adults were members of a club in which they could participate in sport or physical activities, compared to 20.2% of our study population.^11^ However, sports club or organisation membership was higher (23.4%) among those with no long-standing illness or disability. Thus, it may be suggested that future encouragement of sports club membership should include a focus on people with a long-standing illness/disability in order to avoid a potential increase of inequality.

Participants who reported health problems compared to those who did not were less likely to participate in sport at least once over the last year and, in keeping with previous reports,^4–6^ to be physically active fewer days per week. Physical activity participation for all is a key public health goal.^11^ Guidelines have been established to ensure that physical activity participation for those with long-term health conditions is safe and appropriate.^14^ Knowledge of these guidelines by health professionals is essential to ensure that people with long-term health conditions receive appropriate preparticipation and lifelong education regarding safe physical activity. Disadvantaged populations are less knowledgeable about the physical activity guidelines.^27^ ^29^ Brief advice in primary care is a cost-effective way of promoting physical activity^30^ ^31^ and physiotherapists may also have an effective role in assisting people with disabling conditions into physical activity.^32^ Indeed, a rehabilitation intervention, providing tailored sports advice and personalised physical activity counselling, successfully improved physical activity levels in people with acquired physical disabilities in the short^33^ and long^34^ terms.

In keeping with our study findings for the total sample, male adults were more likely to be physically active than females.^6^ Also, no significant association was found between marital status and sport participation in our full sample, but for those with illness/disability, being married/cohabiting (compared to being single) correlated negatively with sport participation. There is debate regarding the association of marital status with physical activity participation.^23^ Also, in keeping with previous reports of physical activity participation in able-bodied adults,^23^ smoking was negatively correlated with sport participation within our full sample but not for those with long-standing illness/disability.

Those reporting no long-standing illness/disability, as for the total sample, were more likely than those who reported such problems to have access to a household car/van and to have educational qualifications, both factors being linked to higher levels of socioeconomic advantage. Not having access to personal transport disadvantages those with a long-standing illness/disability regarding their employment and social opportunities. Indeed, participants reporting no long-term health problems were more likely to report having undertaken paid or unpaid work in the past 7 days, as well as to be of managerial/professional occupational status, and less likely to be socioeconomically disadvantaged. All of these issues may have direct relevance to the influence of a person's social environment on their behaviour and contribute to the lower likelihood of sport participation for those who are less advantaged.

Reporting personal health as ‘fairly good’ and ‘good’ was positively correlated with sport participation in both groups. Asking about a person's health status in the previous year may be an important way to identify those who could benefit from a brief intervention regarding increasing sport and physical activity participation, particularly within clinical practice in primary care.

Doing work was positively associated with sport participation in the full sample and in those with a long-standing illness/disability. Excessive sedentary time is an independent risk factor for adverse health outcomes^35^ and being active is associated with better cardiovascular health and longer life.^36^

Our study showed that living in urban areas was positively correlated with sport participation in both groups, whereas living in more rural areas correlated negatively with sport participation in those with a long-standing illness or disability. This may reflect the variance in infrastructure^37^ and is in keeping with previous reports.^38^ Public health policy planners and commissioners need to consider the potential for creating or exacerbating geographical health inequalities within local communities.^39^ ^40^ We also found that participants reporting a long-standing illness were less likely to have access to the internet, so that the availability of personal internet access should be considered when designing initiatives.

## Strengths and limitations

This study used existing data from CHS, which is representative of the general NI and UK population, with questionnaire respondents being unaware of the focus of this study. Our findings offer insight into an understudied area, reviewing correlates of sport participation in those with a disability or long-standing illness.

A limitation of this study is the potential variation in individuals’ interpretation of the meaning of questions or phrases such as ‘long-term illness/disability’. Other variables which are relevant to sport participation, such as family characteristics,^41^ ^42^ have not been included in our analyses, but such data are not routinely collected or accessible to policy planners. Also, levels of sport participation were not validated externally^6^ and as we only included adults, the results have limited applicability to children.

This is a cross-sectional study, thereby minimising the ability to infer causal relationships between the hypothesised determinants and physical activity. Longitudinal and intervention studies in people with long-term health conditions and sport participation are therefore required.

## Conclusions

Personal (sex, age, marital status), social (educational qualifications, sport club/organisation membership, employment, social class, access to state benefits, level of deprivation) and environmental (car/van access, living in urban or rural locations) barriers and facilitators influence the amount of sport participation people with a disability/long-standing illness undertake. Correlates of sport participation are multifactorial and differ between those in the general adult population and those with long-term illness/disability. Positive correlations for sport participation for the full study population but not in those with a long-standing illness/disability were degree-level education, having internet access and not currently smoking. Negative correlations for sport participation in those with a long-standing illness/disability not found in the full study population included being married or cohabiting compared to being single and greater socioeconomic deprivation.

Health promotion for people with disabilities/long-standing illnesses is an important area for UK policymakers and the health community, particularly in allowing people to meet the current recommended physical activity levels^3^ and tackling the secondary consequences of physical inactivity. The study findings may be utilised in targeting and developing new, tailored public health physical activity and sport participation campaigns for this underserved population group, directing focused efforts towards older females, who are married/cohabiting, socioeconomically deprived, living in rural areas, without personal internet access and who report their health as ‘not good’ in the last year. A further study of initiatives developed to support sport participation among people with long-standing illness/disability is needed.
